# Relationship between remnant cholesterol and short‐term prognosis in acute ischemic stroke patients

**DOI:** 10.1002/brb3.3537

**Published:** 2024-05-07

**Authors:** Zheng Tan, Qianyun Zhang, Qiuwan Liu, Xiaoyin Meng, Wenpei Wu, Long Wang, Juncang Wu

**Affiliations:** ^1^ Department of Neurology Hefei Hospital Affiliated to Anhui Medical University, The Second People's Hospital of Hefei Hefei Anhui China; ^2^ The Fifth Clinical Medical College Anhui Medical University Hefei Anhui China; ^3^ Graduate School Bengbu Medical College Bengbu Anhui China

**Keywords:** acute ischemic stroke, atherosclerotic cardiovascular diseases, low‐density lipoprotein cholesterol, prognosis, remnant cholesterol

## Abstract

**Objective:**

Several studies have illustrated that elevated RC levels are related to a heightened risk of acute ischemic stroke (AIS). Our research aimed to explore the correlation between RC levels and poor prognosis after a 90‐day interval in AIS patients.

**Methods:**

A total of 287 individuals were enrolled in the study, the primary outcome was defined as poor prognosis. RC was derived by the exclusion of low‐density lipoprotein cholesterol (LDL‐C) and high‐density lipoprotein cholesterol (HDL‐C) from total cholesterol (TC).

**Results:**

Following the screening process, 253 AIS patients were included in the study, presenting a median age of 66[57, 75] years. Upon stratifying RC levels into quartiles, those in the top quartile faced a greater likelihood of diabetes diagnosis (42.86%, *p *= .014) and experienced a higher rate of unfavorable outcomes after 90 days (36.51%, *p* = .001). After accounting for confounding factors, the correlation between the fourth quartile of RC levels and the amplified likelihood of poor prognosis remained significant (odds ratio (OR) 8.471, 95% confidence interval (CI) (1.841, 38.985); *p* = .006). Analysis of subgroups unveiled a notable correlation between higher RC levels and poor 90‐day prognosis, particularly in individuals with elevated NIHSS scores (*p* = .044). A progressively increasing 90‐day risk of poor prognosis after an RC greater than 0.38 mmol/L was visualized by restricted cubic spline plots (*p*‐overall = .011).

**Conclusions:**

Including RC as a contributing element may refine the prediction of poor 90‐day prognosis for AIS patients. Integrating RC with traditional risk factors can potentially enhance the predictive value for cerebrovascular disease.

## Graphical abstract

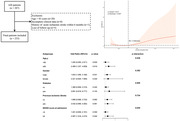

## 1 INTRODUCTION

In light of the expanding and aging population worldwide, there has been a substantial uptick in stroke‐related deaths globally in the recent decade (GBD 2019 Stroke Collaborators, 2021). Specifically, in China, the incidence and death rates from stroke are alarmingly high (Tu, Zhao et al., 2023), with post‐stroke disability and mortality rates standing at 14.8% and 4.2%, respectively (Tu Wang et al., 2023), within 3 months of the event. Investigating the causal factors of stroke has been an enduring endeavor for medical researchers and practitioners, and the dysregulation of lipid metabolism is progressively acknowledged as a substantial contributory element to the pathogenesis of the condition (Holmes et al., 2018).

Low‐density lipoprotein cholesterol (LDL‐C) consistently emerges as a critical factor implicated in the onset and progression of atherosclerotic cardiovascular diseases (ASCVD), including ischemic stroke(Cholesterol Treatment Trialists’ (CTT) Collaboration et al., 2010). Lowering the LDL‐C levels is a core component of ASCVD prevention and treatment.

Remnant cholesterol (RC) is the cholesterol contained in the residues of chylomicron (CM) and very‐low‐density lipoprotein cholesterol (VLDL‐C) after the lipolysis of triglycerides (TG) contained in triglyceride‐rich lipoproteins (TRL) by lipoprotein lipase (LPL). This can be easily obtained by subtracting LDL‐C from total cholesterol (TC) and then subtracting high‐density lipoprotein cholesterol (HDL‐C). Multiple studies have illustrated that heightened RC levels are linked to an elevated risk of ischemic stroke and are independent of risk factors such as LDL‐C (Chen et al., 2021; Gao et al., 2022; Quispe et al., 2021; Shao et al., 2022; Varbo & Nordestgaard, 2019; Wadström et al., 2021). Nevertheless, research into how RC levels influence short‐term prognoses in individuals experiencing acute ischemic stroke (AIS) remains scarce. The present study focused on exploring the association between RC levels and poor prognosis of AIS patients at 90 days, hoping to provide more precise and individualized treatment for patients.

## 2 MATERIALS AND METHODS

### 2.1 Study design

In the course of this retrospective study, we continuously recruited patients between the ages of 18 and 85 with acute ischemic stroke who were hospitalized between July 2022 and June 2023 at the Hefei Hospital Affiliated with Anhui Medical University. We gathered information concerning patient demographics, clinical diagnoses, laboratory tests, and imaging findings during hospitalization and assessed the degree of neurological impairment, quality of life, and functional status. Our research aimed to explore the correlation between RC levels and poor prognosis after a 90‐day interval in patients diagnosed with AIS.

### 2.2 Population

**FIGURE 1:**
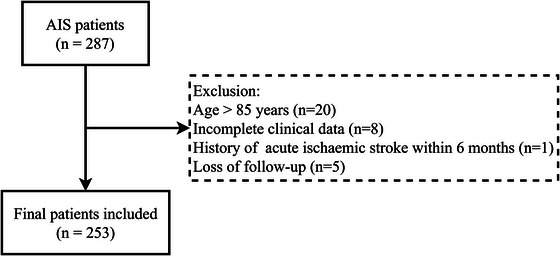
Flowchart of the study population.

The study encompassed a cohort of 287 participants. To ensure the reliability and validity of our research, we excluded the following individuals: (1) age greater than 85 years or age less than 18 years; (2) had a history of acute ischemic stroke within 6 months; (3) had pre‐existing serious brain disease; (4) diagnosed of cerebral hemorrhage, severe hepatic and renal insufficiency, or hematologic and oncologic diseases; (5) had insufficient clinical information or lost to follow‐up. The final study population comprised 253 individuals; among the exclusions, 20 were due to age exceeding 85 years, 8 had insufficient clinical information, 1 had a history of AIS within the past 6 months, and 5 were lost to follow‐up (Figure 1). The primary reasons for the loss to follow‐up were patient refusal or inability to contact.

### 2.3 Data collection

Demographic characteristics and clinical data were obtained from hospitalized patients, such as age, gender, lifestyle habits (smoking, alcohol consumption), body mass index (BMI), history of previous diseases (diabetes mellitus, hypertension, coronary heart disease [CHD], ischemic stroke), whether or not they were treated with intravenous thrombolysis or endovascular thrombectomy, and laboratory results under fasting conditions within 24 h of admission to the hospital. AIS was diagnosed based on the signs and symptoms on admission and diffusion‐weighted magnetic resonance imaging (DWI) scans, consistent with the International Classification of Diseases 11th edition (ICD‐11) disease codes. The degree of neurologic impairment in AIS patients was quantified through the application of the National Institutes of Health Stroke Scale (NIHSS) score (Kasner, 2006). The patients’ functional status and quality of life were assessed using the modified Rankin scale (mRS) score at the 90‐day follow‐up (Kasner, 2006), typically conducted by telephone or by visiting the clinic.

### 2.4 Measurement of blood lipids

Laboratory data on TG and TC levels were obtained using enzymatic assays. The HDL‐C and LDL‐C levels were measured directly. VLDL‐C was derived using a calculated method. RC was calculated using the formula: TC minus LDL‐C and HDL‐C (Jacobson et al., 2015). To comprehensively enhance our understanding of the distribution of RC levels, we calculated the quartiles of the data at 25%, 50%, and 75% to obtain the quartile range for RC.

### 2.5 Clinical outcome

Early neurologic deterioration (END), as outlined in pertinent studies, was defined as an increase of ≥2 points in the overall NIHSS score or an increase of ≥1 point in consciousness or motor scores of the NIHSS score within 72 h of onset (Chang et al., 2021; Jeong et al., 2015). The mRS score at 90 days was determined by a specialist neurologist during follow‐up. The score of mRS ≤2 represents a good prognosis (0 to 2), and mRS > 2 represents a poor prognosis (3 to 6), with 6 representing death (Quinn et al., 2009; van Swieten et al., 1988). The primary endpoint was specified as a poor prognosis.

### 2.6 Statistical analysis

Data analysis was conducted using the SPSS (version 25.0; IBM Corp., USA) and R (version 4.3.2; R Foundation for Statistical Computing, Austria) software. For continuous variables, data conforming to a normal distribution were expressed by calculating the mean and standard deviation, those not adhering to normality were represented by the median and quartile values. Comparisons between groups were made using ANOVA variance and Kruskal–Wallis tests. Categorical data are presented in terms of their frequencies and corresponding percentages, with the chi‐square test being utilized to assess intergroup variability. Fisher's exact test was used for subgroups with lower expected frequencies to ensure the accuracy of the test. The effect of each variable on functional prognosis was analyzed using univariate logistic regression, and the relationship between RC levels and 90‐day functional prognosis was further analyzed by adjusting for confounders for variables with *p* < .1. To identify the possible influencing factors in greater detail, subgroup analyses were carried out to verify the reliability and usefulness of the findings. Restricted cubic spline plots were used to provide a clearer and more intuitive prediction of the relationship between RC and poor prognosis, with the number of knots set to four to fit the spline function (5th, 35th, 65th, and 95th percentiles). Statistical significance was defined as a two‐sided *p*‐value of < .05.

## 3 RESULTS

### 3.1 Characteristics of the study population

As shown in Table 1, a cohort of 253 patients with AIS was established following screening. The median age within this group was 66 [57, 75] years, and 32.41% were female. Following the stratification of RC levels into quartiles, no significant variations were observed in age or gender across groups (*p *> .05). Similarly, no noteworthy differences were observed across the groups in hypertension, CHD, atrial fibrillation, smoking, alcohol consumption, BMI, intravenous thrombolysis therapy, thrombectomy, or END factors (*p* > .05). In contrast, over one‐fifth of the enrolled patients (54, 21.34%) were categorized as having a poor prognosis during follow‐up, while those in the fourth quartile group expressed an increased probability of receiving a diabetes diagnosis (42.86%, *p *= .014) and demonstrated a greater prevalence of poor prognosis at 90 days (36.51%, *p* = .001) than the other subgroups. In addition, individuals in the fourth quartile group had higher levels of TG (2.050 [1.490, 3.240] mmol/L), TC (5.380 [4.835, 5.980] mmol/L), LDL (3.530 [3.020, 4.210] mmol/L), and VLDL (0.410 [0.300, 0.645] mmol/L) compared to the other three groups, which was significantly different (*p* < .001).

**TABLE 1:** Characteristics of participants stratified by remnant cholesterol levels.

		Quartile grouping of remnant cholesterol				
	Overall (*n* = 253)	1st quartile (*n* = 64)	2nd quartile (*n* = 66)	3rd quartile (*n* = 60)	4th quartile (*n* = 63)	*p* for trend
		(< = 0.27 mmol/L)	(0.27 to < = 0.38 mmol/L)	(0.38 to < = 0.51 mmol/L)	(> 0.51 mmol/L)	
Gender, *n* (%)						
Female	82 (32.41)	14 (21.88)	24 (36.36)	22 (36.67)	22 (34.92)	0.222
Male	171 (67.59)	50 (78.12)	42 (63.64)	38 (63.33)	41 (65.08)	
Age, years (median [IQR])	66 [57, 75]	66 [58, 76]	68 [57, 75]	67 [60, 73]	65 [54, 74]	0.628
Hypertension, *n* (%)	176 (69.57)	47 (73.44)	42 (63.64)	40 (66.67)	47 (74.60)	0.468
Diabetes, *n* (%)	81 (32.02)	17 (26.56)	13 (19.70)	24 (40.00)	27 (42.86)	0.014 *
Previous ischemic stroke, *n* (%)	84 (33.20)	31 (48.44)	17 (25.76)	14 (23.33)	22 (34.92)	0.011 *
CHD, *n* (%)	19 (7.51)	4 (6.25)	5 (7.58)	1 (1.67)	9 (14.29)	0.064
Atrial fibrillation, *n* (%)	18 (7.11)	7 (10.94)	5 (7.58)	3 (5.00)	3 (4.76)	0.499
Smoking, *n* (%)	70 (27.67)	21 (32.81)	19 (28.79)	11 (18.33)	19 (30.16)	0.296
Alcohol, *n* (%)	58 (22.92)	20 (31.25)	11 (16.67)	12 (20.00)	15 (23.81)	0.232
BMI (median [IQR])	23.88 [22.04, 25.88]	23.98 [22.21, 26.07]	23.77 [21.99, 26.02]	23.86 [21.96, 25.87]	23.88 [22.10, 25.45]	0.984
Intravenous thrombolysis, *n* (%)	35 (13.83)	10 (15.62)	11 (16.67)	7 (11.67)	7 (11.11)	0.742
Thrombectomy, *n* (%)	6 (2.37)	2 (3.12)	1 (1.52)	3 (5.00)	0 (0.00)	0.297
NIHSS score at admission (median [IQR])	3 [2, 7]	4 [2, 9]	3 [1, 5]	3 [1, 5]	3 [2, 8]	0.117
END, *n* (%)	26 (10.28)	8 (12.50)	6 (9.09)	4 (6.67)	8 (12.70)	0.639
mRS score at 90 days > 2, *n* (%)	54 (21.34)	16 (25.00)	7 (10.61)	8 (13.33)	23 (36.51)	0.001 **
TG, mmol/L (median [IQR])	1.340 [1.010, 1.890]	1.055 [0.768, 1.290]	1.150 [0.952, 1.445]	1.415 [1.138, 1.837]	2.050 [1.490, 3.240]	<0.001 ***
TC, mmol/L (median [IQR])	4.480 [3.680, 5.230]	3.500 [3.050, 4.400]	4.205 [3.688, 4.525]	4.825 [4.220, 5.640]	5.380 [4.835, 5.980]	<0.001 ***
HDL‐C, mmol/L (median [IQR])	1.100 [0.960, 1.320]	1.055 [0.918, 1.252]	1.105 [0.970, 1.360]	1.235 [0.978, 1.382]	1.130 [0.940, 1.260]	0.079
LDL‐C, mmol/L (median [IQR])	2.890 [2.330, 3.530]	2.295 [1.765, 2.967]	2.640 [2.212, 3.058]	3.120 [2.717, 3.907]	3.530 [3.020, 4.210]	<0.001 ***
VLDL‐C, mmol/L (median [IQR])	0.270 [0.200, 0.380]	0.210 [0.150, 0.260]	0.230 [0.192, 0.290]	0.280 [0.228, 0.365]	0.410 [0.300, 0.645]	<0.001 ***
RC, mmol/L (median [IQR])	0.380 [0.270, 0.510]	0.215 [0.170, 0.240]	0.335 [0.310, 0.360]	0.440 [0.410, 0.480]	0.630 [0.580, 0.875]	<0.001 ***

Abbreviations: CHD, coronary heart disease; BMI, body mass index; NIHSS, National Institutes of Health Stroke Scale; END, early neurological deterioration; mRS, modified Rankin Scale; TG, triglycerides; TC, total cholesterol; HDL‐C, high‐density lipoprotein cholesterol; LDL‐C, low‐density lipoprotein cholesterol; VLDL‐C, very low‐density lipoprotein cholesterol; RC, remnant cholesterol; IQR, interquartile range.

*p < .05, ** *p* < .01, *** *p *< .001.

### 3.2 Univariate and multivariate analysis

When analyzing the effect of various factors on poor prognosis, it was found that age, diabetes mellitus, previous ischemic stroke, coronary heart disease, thrombectomy therapy, NIHSS score at admission, END, and HDL‐C were correlated with a higher likelihood of poor prognosis (odds ratio [OR] 1.029, 95% confidence interval [95% CI] [1.001, 1.057]; *p* = .046, OR 2.431, 95% CI [1.311, 4.509]; *p* = .005, OR 2.491, 95% CI [1.346, 4.611]; *p* = .004, OR 3.780, 95% CI [1.451, 9.847]; *p* = .006, OR 20.204, 95% CI [2.308, 176.898]; *p *= .007, OR 1.343, 95% CI [1.236, 1.459]; *p* < .001, OR 11.937, 95% CI [4.826, 29.531]; *p *< .001, OR 0.270, 95% CI [0.083, 0.876]; *p *= .029), and in RC quartiles, with the second quartile as a reference, the level of RC in the fourth quartile (OR 4.846; 95% CI [1.900, 12.362]; *p *= .001) was significantly correlated with poor 90‐day prognosis (Table 2). Upon making adjustments after adding the confounding factors of atrial fibrillation, the association of NIHSS score at admission, and END with poor prognosis at 90 days remained significant (OR 1.373, 95% CI [1.226, 1.538]; *p *< .001; OR 36.224, 95% CI [9.499, 138.132]; *p* < .001), and the level of RC in the fourth quartile (OR 8.471, 95% CI [1.841, 38.985]; *p *= .006) also showed an increased likelihood of poor prognosis at 90 days.

**TABLE 2:** Univariate analysis and multivariate analyses of patients with acute ischemic stroke and poor outcomes.

Variable	Univariate analysis				Multivariate analysis	
	mRS (0–2)	mRS (3–6)	OR (95% CI)	*p* Value	Adjusted OR (95% CI)	*p* Value
Gender (Male), *n* (%)	134 (67.34)	37 (68.52)	1.056 (0.553, 2.015)	.869		
Age, years (median [IQR])	66 [57, 74]	67 [60, 78]	1.029 (1.001, 1.057)	.046 *	1.028 (0.983, 1.074)	.224
Hypertension, *n* (%)	135 (67.84)	41 (75.93)	1.495 (0.749, 2.984)	.254		
Diabetes, *n* (%)	55 (27.64)	26 (48.15)	2.431 (1.311, 4.509)	.005 **	2.590 (0.944, 7.106)	.065
Previous Ischemic stroke, *n* (%)	57 (28.64)	27 (50.00)	2.491 (1.346, 4.611)	.004 **	1.289 (0.473, 3.515)	.620
CHD, *n* (%)	10 (5.03)	9 (16.67)	3.780 (1.451, 9.847)	.006 **	2.665 (0.502, 14.150)	.250
Atrial fibrillation, *n* (%)	11 (5.53)	7 (12.96)	2.545 (0.936, 6.920)	.067	0.180 (0.023, 1.388)	.100
Smoking, *n* (%)	56 (28.14)	14 (25.93)	0.894 (0.452, 1.769)	.747		
Alcohol, *n* (%)	47 (23.62)	11 (20.37)	0.827 (0.395, 1.732)	.615		
BMI (median [IQR])	23.81 [22.03, 25.85]	24.14 [22.49, 25.91]	1.029 (0.951, 1.115)	.473		
Intravenous thrombolysis, *n* (%)	24 (12.06)	11 (20.37)	1.865 (0.848, 4.101)	.121		
Thrombectomy, *n* (%)	1 (0.50)	5 (9.26)	20.204 (2.308, 176.898)	.007 **	6.418 (0.445, 92.620)	.172
NIHSS score at admission (median [IQR])	3 [1, 4]	10 [6, 14]	1.343 (1.236, 1.459)	<.001 ***	1.373 (1.226, 1.538)	<.001 ***
END, *n* (%)	8 (4.02)	18 (33.33)	11.937 (4.826, 29.531)	<.001 ***	36.224 (9.499, 138.132)	<.001 ***
TG, mmol/L (median [IQR])	1.34 [1.01, 1.89]	1.28 [1.03, 2.00]	0.895 (0.653, 1.228)	.492		
TC, mmol/L (median [IQR])	4.43 [3.74, 5.19]	4.62 [3.46, 5.44]	1.035 (0.797, 1.343)	.798		
HDL‐C, mmol/L (median [IQR])	1.13 [0.97, 1.36]	1.06 [0.89, 1.25]	0.270 (0.083, 0.876)	.029 *	0.723 (0.126, 4.152)	.716
LDL‐C, mmol/L (median [IQR])	2.86 [2.36, 3.45]	3.09 [2.12, 3.64]	1.021 (0.749, 1.391)	.896		
VLDL‐C, mmol/L (median [IQR])	0.27 [0.20, 0.38]	0.26 [0.21, 0.40]	0.581 (0.120, 2.804)	.499		
RC (quartiles) (%)						
< = 0.27 mmol/L	48 (24.12)	16 (29.63)	2.810 (1.069, 7.385)	.036 *	2.804 (0.624, 12.601)	.179
0.27 to < = 0.38 mmol/L	59 (29.65)	7 (12.96)	Reference			
0.38 to < = 0.51 mmol/L	52 (26.13)	8 (14.81)	1.297 (0.440, 3.821)	.637	1.562 (0.258, 9.436)	.627
>0.51 mmol/L	40 (20.10)	23 (42.59)	4.846 (1.900, 12.362)	.001 **	8.471 (1.841, 38.985)	.006 **

Abbreviations: CHD, coronary heart disease; BMI, body mass index; NIHSS: National Institutes of Health Stroke Scale; END, early neurological deterioration; TG, triglycerides; TC, total cholesterol; HDL‐C, high‐density lipoprotein cholesterol; LDL‐C, low‐density lipoprotein cholesterol; VLDL‐C, very low‐density lipoprotein cholesterol; RC, remnant cholesterol; mRS, modified Rankin Scale; IQR, interquartile range; OR, odds ratio; CI, confidence interval.

* *p* < .05, ** *p* < .01, *** *p* < .001.

### 3.3 Subgroup analysis and restricted cubic spline

**FIGURE 2:**
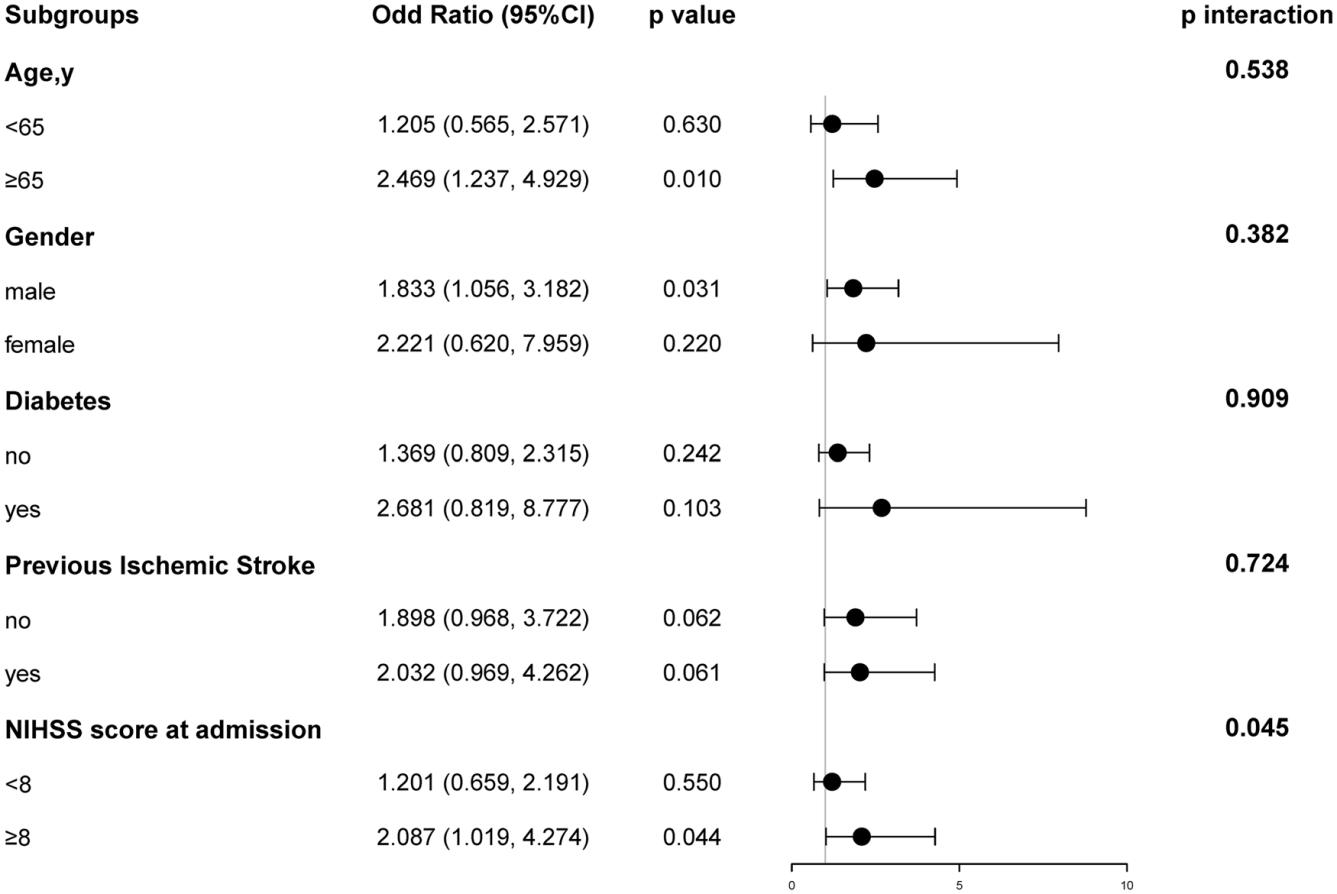
Subgroup analysis of the relationship between remnant cholesterol (RC) levels and 90‐day prognosis. Adjusted for age, gender, hypertension, diabetes mellitus, previous ischemic stroke, coronary heart disease, atrial fibrillation, smoking, alcohol, BMI, intravenous thrombolysis therapy, thrombectomy therapy, National Institutes of Health Stroke Scale (NIHSS) score at admission, and early neurological deterioration (END) variables.

Further subgroup analysis of age (< 65 or ≥65 years), gender, diabetes mellitus, previous ischemic stroke, and NIHSS scores at admission (< 8 or ≥8 scores) (Figure 2) showed that in the age ≥65 years group, RC level was significantly linked to poor prognosis (OR 2.469, 95% CI [1.237, 4.929]; *p* = .010) in a positive manner, whereas in the age < 65 years subgroup, statistical significance was not reached by the observed correlation (OR 1.205, 95% CI [0.565, 2.571]; *p* = .630). Likewise, in the gender‐specific subgroup analysis, a significant meaningful correlation was observed between male patients and their adverse prognosis (OR 1.833, 95% CI [1.056, 3.182]; *p* = .031). Interestingly, for the subgroup presenting NIHSS score < 8 on admission, RC levels were not significantly associated with 90‐day adverse prognosis (OR 1.201, 95% CI [0.659, 2.191]; *p* = .550), whereas a noticeable correlation was demonstrated in the subgroup with NIHSS score ≥8; the OR was significantly increased to 2.087, with a *p*‐value of.044. Furthermore, in the analysis of each specific subgroup, barring the one differentiated by NIHSS scores (*p* = .045), there were no detectable statistical distinctions discernible within the subgroups (*p *> .05).

**FIGURE 3:**
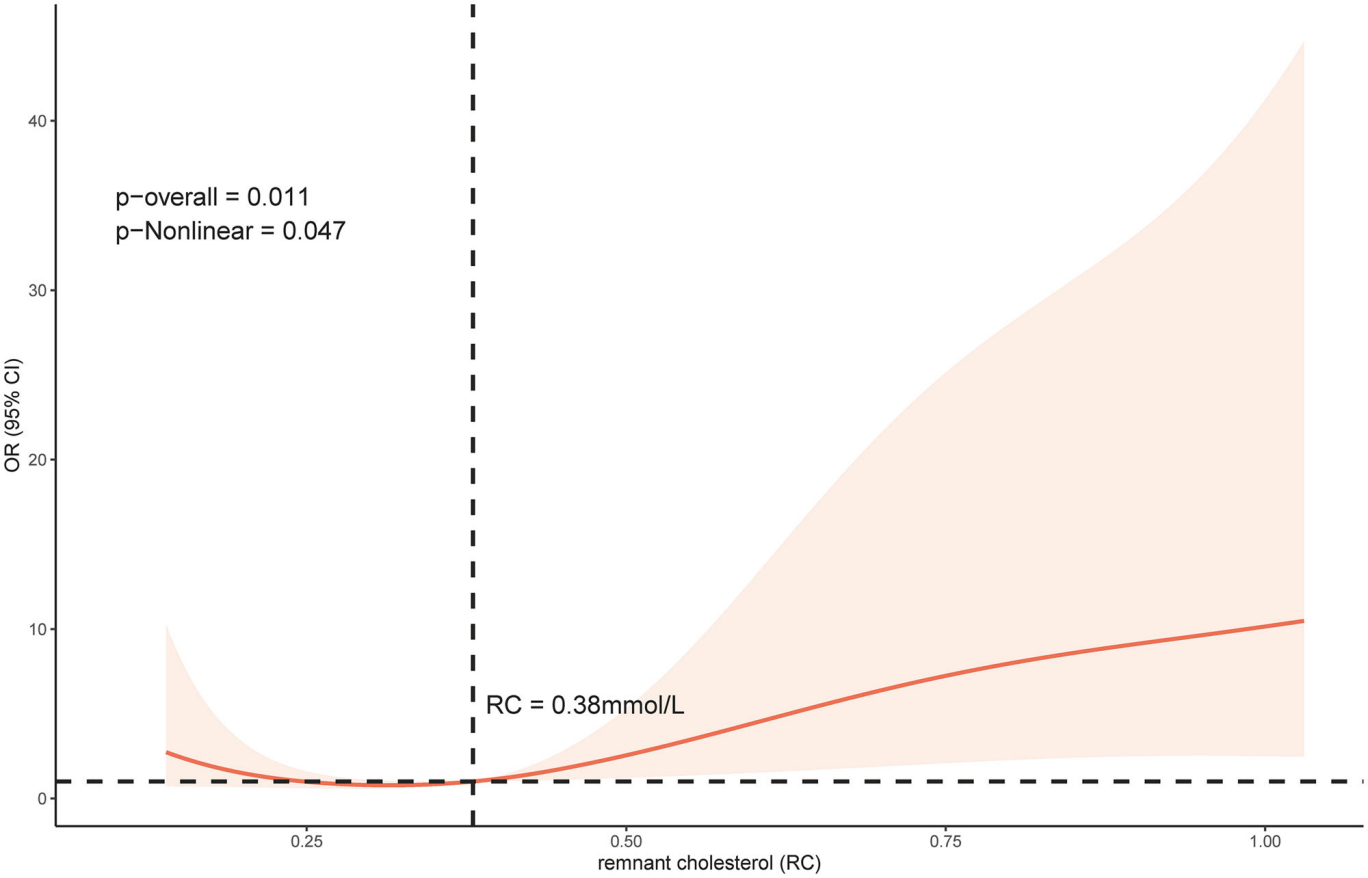
Restricted cubic spline. Adjusted for age, diabetes mellitus, previous ischemic stroke, coronary heart disease, atrial fibrillation, smoking, intravenous thrombolysis therapy, thrombectomy therapy, National Institutes of Health Stroke Scale (NIHSS) score at admission, early neurological deterioration (END), and high‐density lipoprotein cholesterol (HDL‐C) variables.

To provide a clearer and more intuitive prediction of the relationship between RC and poor prognosis, we used logistic regression analysis to construct restricted cubic spline plots, adjusting for age, diabetes mellitus, previous ischemic stroke, coronary heart disease, atrial fibrillation, smoking, intravenous thrombolysis therapy, thrombectomy therapy, NIHSS score at admission, END, HDL‐C, as confounding variables. As shown in Figure 3, the model exhibited a non‐linear correlation (*p*‐nonlinear = .047) between RC levels and the risk of unfavorable prognosis after 90 days, and the likelihood of a poor 90‐day prognosis was relatively flat until the RC level was 0.38 mmol/L. Then, the risk gradually increased after this threshold (*p*‐overall = .011).

## 4 DISCUSSION

In this study, we ultimately enrolled 253 patients hospitalized for AIS, and poor prognosis at 90 days was correlated with higher NIHSS scores on admission, END events, and elevated RC levels. The observed correlations remained statistically significant upon adjusting for potential confounders, including age, diabetes mellitus, previous ischemic stroke, CHD, atrial fibrillation, thrombectomy therapy, and HDL‐C. Within the examined subgroups, the unfavorable prognosis was significantly linked to individuals older than 65 years, male gender, and an admission NIHSS score of 8 or above. Upon examining subgroups delineated by NIHSS scores, considerable distinctions were observed between the two groups, which implies a possible correlation between the RC levels and poorer 90‐day mRS results, particularly in individuals with elevated NIHSS scores. Meanwhile, an escalating risk for adverse outcomes was observed in correlation with rising RC levels, particularly when levels surpassed the threshold of 0.38 mmol/L.

Multiple research endeavors have scrutinized the link between RC levels and the initiation of AIS, uncovering noteworthy correlations. In a prospective study based on a general population of 112,512 people (Varbo & Nordestgaard, 2019), the Copenhagen General Population Study, encompassing 102,964 participants, suggested that increased RC levels were associated with a heightened likelihood of experiencing ischemic stroke. The correlation remained significant after sensitivity analyses, which was further corroborated through a longitudinal follow‐up of 9548 participants in the Copenhagen City Heart Study spanning up to 26 years. Another longitudinal investigation, also based on the Copenhagen General Population Study and the Copenhagen City Heart Study, showed that increased RC levels were correlated with a heightened risk for stroke as indicated by hazard ratios (HR) of 1.8 (95% CI 1.4–2.5) and 2.1 (95% CI 1.5–3.1)(Wadström et al., 2021).

The community‐level disease prevention study conducted by Quispe et al., totaling 9748 individuals, also indicated that increased RC levels were correlated with the onset of ASCVD, regardless of LDL‐C and apolipoprotein B (ApoB) levels (Quispe et al., 2021). A cohort study carried out on the Korean national population including 956,452 patients with diabetes demonstrated a heightened risk of ischemic stroke linked to increased RC levels (HR 1.22, 95% CI [1.195–1.247]), and the results remained significant after stratified analysis (Huh et al., 2022). A meta‐analysis incorporating 31 studies(Yang et al., 2023) revealed that elevated RC levels were significantly linked to a heightened likelihood of ischemic stroke compared to low levels of RC (relative risk [RR] 1.43, 95% CI [1.24–1.66]).

Recent studies also support similar viewpoints. A follow‐up study involving 6764 individuals revealed a robust positive link between elevated RC levels and stroke risk (HR 1.87, 95% CI [1.08–3.25]; *p *= .034) (Liu et al., 2023). A longitudinal study of 10,067 middle‐aged and elderly people in China showed a significant link between RC levels and a heightened risk of stroke (HR 1.087, 95% CI [1.001–1.180]). This correlation was nonlinear, with the most pronounced connection occurring at RC levels under 1.78 mmol/L (Wang et al., 2023). In a 6‐year follow‐up of a 7035‐person cohort, elevated RC was linked to a heightened stroke risk (Li et al., 2023), persistent even after controlling for various influencing factors (OR 1.74, 95% CI [1.06−2.85]). The research pointed to a notable intermediary role of hypertension in the RC–stroke relationship, unlike diabetes, which did not demonstrate a significant mediation, somewhat diverging from our study results. Moreover, a recent study looking back at AIS patients who received mechanical thrombectomy showed that increased RC levels correlate with negative prognoses in patients without large artery atherosclerosis (Li et al., 2024). Our research contributes additional information to this discovery.

Mechanistically, the correlation between increased RC levels and a high likelihood of stroke can be accounted for by RC‐induced atherosclerosis (Nordestgaard & Varbo, 2014). Lipids infiltrate and become ensnared within the arterial wall, setting the stage for cholesterol deposition in the arterial lining and ultimately leading to atherosclerosis. Lipoproteins of different sizes have different chances of entering and becoming trapped in the arterial walls. In comparison to LDL‐C, RC demonstrates a relatively larger size, which facilitates their entrapment within the arterial intima subsequent to arterial wall penetration (Nordestgaard, 2016; Shaikh et al., 1991). This process results in cholesterol accumulation within the intima and contributes to the pathogenesis of atherosclerosis, potentially culminating in ischemic stroke (Li et al., 2022). In vitro studies have shown that RC can be directly taken up by macrophages (Nakajima et al., 2006) and that RC particles possess a larger size and a higher cholesterol‐carrying capacity than LDL‐C particles (Salinas & Chapman, 2020); therefore, it has a greater atherogenic capacity when it enters the endothelium. Other factors such as inflammation (Bernelot Moens et al., 2017; Lugonja et al., 2023; Varbo et al., 2013), induction of endothelial dysfunction (Liu et al., 2009), and vascular endothelial injury (Packard, 2022) may also be potential pathogenic mechanisms of RC.

Within this research, similar to several large‐scale cohort studies, indirect calculations were used to derive the RC (Langsted et al., 2020; Varbo, Benn, Tybjærg‐Hansen, Jørgensen et al., 2013). Notably, venous blood collection in China is mostly done in the early morning of the second day after admission, except for emergency blood tests. Multiple studies have indicated that, whether in fasting or non‐fasting states, RC is correlated with inflammation, oxidative stress, and the progression of atherosclerosis (Liu et al., 2009; Zheng & Liu, 2007).

Following the protocols recommended by Chinese health authorities for stroke intervention and secondary prevention, statins are routinely prescribed and taken by most individuals for both preventative and therapeutic uses. Statins have been shown to exert a positive influence on the occurrence of ischemic stroke (Amarenco & Labreuche, 2009; Cholesterol Treatment Trialists’ (CTT) Collaboration et al., 2010; Cholesterol Treatment Trialists’ (CTT) Collaborators, 2012), as well as in lowering TG and RC (Karlson et al., 2016; Miller et al., 2016; Puri et al., 2016; Tsujita et al., 2016; Varbo & Nordestgaard, 2017; Würtz et al., 2016); however, a residual risk persists. Mendelian randomization studies have shown that statins, proprotein convertase subtilisin/kexin type 9 (PCSK9) inhibitors, ezetimibe, and angiopoietin‐like protein 3 can reduce RC and LDL‐C (Tikkanen et al., 2019; Wang et al., 2021). Several studies also depict that ezetimibe combined with statins results in a more substantial reduction in RC (Ahmed et al., 2018; Mangili et al., 2014). However, no significant benefit has been shown to be achieved by reducing RC levels. Currently, the main principles of management for elevated RC levels are lifestyle changes and correction of secondary factors.

In this study, we leverage insights from several large‐scale cohort studies to explore the connection between RC levels and the incidence of poor outcomes after 90 days. In addition, the application of calculations to derive RC from lipid profiles is both convenient and cost‐effective, making it more universally applicable. However, there are still drawbacks: The first one to note is that this study is a single‐center study, which may lead to some bias in terms of geographic, demographic, and medical practice. Second, logistic regression analysis in this study yielded no substantial evidence of a relationship between LDL‐C and 90‐day poor prognosis; unfortunately, no further comparative analysis with RC is possible, which needs to be further expanded to study the sample size in‐depth. Third, the present study used fasting blood samples obtained after admission, and it may be more persuasive if the average level of RC is calculated by dynamically collecting the blood samples multiple times.

## 5 CONCLUSION

Including RC as a contributing element may refine the prediction of poor 90‐day prognosis for AIS patients. Integrating RC with traditional risk factors can potentially enhance the predictive value for cerebrovascular disease.

## AUTHOR CONTRIBUTIONS

**Juncang Wu** and **Long Wang**: Conceptualization; funding acquisition; supervision; writing—review and editing. **Zheng Tan**: Conceptualization; data curation; formal analysis; investigation; methodology; software; writing—original draft. **Qianyun Zhang**: Data curation; formal analysis; investigation; validation. **Qiuwan Liu**: Formal analysis; investigation; methodology; validation. **Xiaoyin Meng** and **Wenpei Wu**: Formal analysis; investigation. All authors have read and endorsed the submission and publication of this manuscript.

## CONFLICT OF INTEREST STATEMENT

No conflicts of interest are declared by any of the authors in this study.

### PEER REVIEW

The peer review history for this paper is available at https://publons.com/publon/10.1002/brb3.3537

## Data Availability

Data supporting the findings of this study can be furnished by the corresponding author, subject to a justified request.
